# Efficacy of Adjuvant Chemotherapy for T1-2 Stage III Colorectal Cancer

**DOI:** 10.3390/cancers17233856

**Published:** 2025-11-30

**Authors:** Hiromichi Maeda, Koji Oba, Kosuke Kashiwabara, Toru Aoyama, Shuhei Mayanagi, Mitsuro Kanda, Michitaka Honda, Masaru Muto, Junichi Sakamoto, Hisakazu Yamagishi, Takaki Yoshikawa

**Affiliations:** 1 Department of Surgery, Kochi Medical School, Nankoku 783-8505, Japan; 2 Department of Biostatistics, Graduate School of Medicine, The University of Tokyo, Tokyo 113-0033, Japan; 3 Data Science Office, Clinical Research Promotion Center, The University of Tokyo Hospital, Tokyo 113-8655, Japan; 4 Department of Surgery, Yokohama City University, Yokohama 236-0004, Japan; 5 Division of Esophageal Surgery, Shizuoka Cancer Center Hospital, Shizuoka 411-8777, Japan; 6 Department of Gastroenterological Surgery, Nagoya University Graduate School of Medicine, Nagoya 466-8550, Japan; 7 Department of Minimally Invasive Surgical and Medical Oncology, Fukushima Medical University, Fukushima 960-1247, Japan; 8 Japanese Foundation for Multidisciplinary Treatment of Cancer, Tokyo 136-0071, Japan; 9 Tokai Central Hospital, Kakamigahara 504-8601, Japan; 10 Department of Gastric Surgery, National Cancer Center Hospital, Tokyo 104-0045, Japan

**Keywords:** colorectal cancer, adjuvant chemotherapy, T1, T2

## Abstract

**Background/Objectives**: This study aimed to assess the efficacy of adjuvant chemotherapy for T1-2 stage III colorectal cancer, a disease with a low recurrence rate. **Methods**: The efficacies of fluorouracil-based adjuvant chemotherapy (5FU group) and oxaliplatin-based adjuvant chemotherapy (L-OHP group) were assessed and compared with that of surgery alone (surgery group) using data from seven clinical trials conducted by the Japanese Foundation for Multidisciplinary Treatment of Cancer. Propensity score matching was used to compare the three groups. Direct-adjusted survival curves were delineated with consideration of treatment periods. **Results**: A total of 604 patients with T1-2 stage III colorectal cancer were identified. After adjusting for the patient factors, the hazard ratio of relapse-free survival (RFS) was 0.79 (95% confidence interval (CI): 0.13–4.65, *p* = 0.79) and 0.64 (95% CI: 0.06–6.40, *p* = 0.70) in the 5FU and L-OHP groups, respectively. Adjusted 5-year RFS rate was 82.8% (95% CI: 67.2–100%), 86.2% (95% CI: 74.2–100%), and 88.6% (95% CI: 74.0–100%) in the surgery, 5-FU, and L-OHP groups, respectively. Overall and disease-specific survival showed similar trends without significant differences. **Conclusions**: No significant improvement in prognosis was observed after adjuvant chemotherapy. The potential improvement in the 5-year RFS after adjuvant chemotherapy for resected T1-2 stage III colorectal cancer should be balanced with patient factors and adverse events.

## Simple Summary

Stage III colorectal cancer is a heterogeneous group of cases. In this study, we retrospectively examined the effects of postoperative chemotherapy on T1-2 stage III colorectal cancer, which is considered to have a favorable prognosis. A total of 604 eligible cases were extracted from a database of previous clinical trials and analyzed. After adjusting for the patient factors, the hazard ratio of relapse-free survival (RFS) was 0.79 (95% confidence interval (CI): 0.13–4.65, *p* = 0.79) and 0.64 (95% CI: 0.06–6.40, *p* = 0.70) in the 5FU and L-OHP groups, respectively. Postoperative chemotherapy showed a better prognosis than surgery alone, but the margin of improvement was minute. The results of this study are useful for decision-making regarding postoperative chemotherapy for T1-2 stage III colorectal cancer.

## 1. Introduction

Adjuvant chemotherapy with a fluoropyrimidine-based regimen (Fluorouracil [5FU]) has been administered after curative surgical resection of colorectal cancer in order to reduce the risk of recurrence [1,2,3]. A pooled analysis of 3202 patients with stage II and III colon cancer who underwent curative resection exhibited a 5-year disease-free survival (DFS) rate of 55% with no further treatment and 67% after 5FU, with a proportional risk reduction of 30% [2]. Furthermore, adding oxaliplatin to 5FU further reduced the 5-year cumulative recurrence rate by 5% [4].

Resected stage III and high-risk stage II colon cancers are currently targeted for postoperative adjuvant chemotherapy [5,6,7]. The treatment regimen for pathologically diagnosed stage III rectal cancer also includes adjuvant chemotherapy [8,9,10]. However, patients with stage III colorectal cancer are not a homogeneous but rather a heterogeneous population with widely varying prognoses. Patients with node-positive T4 colon cancer have approximately a 5-year overall survival (OS) rate of 60–80% [11]. Meanwhile, patients with node-positive stage IIIa T1/2 colon cancer exhibited a far better OS rate (70–90%) than those with T4 Stage II colon cancer [12,13].

The magnitude of the benefit induced by adjuvant chemotherapy may differ according to the prognosis at baseline. Therefore, the use of adjuvant chemotherapy for T1 or T1-2 stage III colorectal cancer remains debatable [2,14,15,16]. Notably, some researchers have reported significant and profound benefits [14], whereas others have expressed contrasting views on adjuvant chemotherapy in patients with T1-2 stage III colorectal cancer [15]. Because the results of these studies are accompanied by the possible bias of registered databases or single-center experience, we aimed to assess the efficacies of oral 5FU and oxaliplatin-based chemotherapy (L-OHP) in patients with T1-2 stage III colorectal cancer using pooled data from clinical trials.

## 2. Materials and Methods

### 2.1. Patients and Data

This study used data from clinical trials conducted by the Japanese Foundation for Multidisciplinary Treatment of Cancer (JFMC). JFMC 07, 15, and 38 had investigated the efficacy of oral 5FU compared with that of surgery alone [1]. JFMC 33, 35, and 37 examined the optimal dosing schedule of oral 5FU [17,18,19], whereas JFMC 41 focused on the safety of L-OHP in an exploratory study of prognosis after treatment [20]. The patient accrual periods for each study were as follows: JFMC07 (February 1986–December 1988), JFMC15 (January 1989–December 1990), JFMC33 (October 2005–September 2007), JFMC35 (April 2006–March 2009), JFMC37 (September 2008–December 2009), JFMC38 (October 2011–February 2013), and JFMC41 (November 2010–March 2012).

Patients with T1-2 node-positive colorectal cancer who underwent D2/3 dissection were included in this study. Patients with missing data and/or D1 lymph node dissection were excluded from the analysis. The extent of lymph node dissection in colorectal cancer is outlined below. Lymph nodes targeted for dissection are classified into three groups: pericolic/perirectal nodes, intermediate, and main lymph nodes [21]. Pericolic/perirectal nodes refer to those located adjacent to the intestine. For the colon, the general principle is to resect approximately 10 cm of bowel longitudinally on both sides of the tumor, whereas for the rectum, the standard is to resect 2–3 cm toward the anus. The appropriateness of these resection lengths is supported by that lymph node metastasis beyond this range is rare [22,23,24].

Intermediate lymph nodes are located along the supplying vessels, while main lymph nodes are situated at the root of these vessels [21]. Dissection of all three groups is defined as D3 dissection, whereas removal of only paracolic and intermediate lymph nodes is classified as D2 dissection. These extents of dissection are supported by the presence of metastasis in intermediate and main lymph nodes, as well as differences in postoperative survival rates in T3/T4 disease [25].

The study protocol was approved by the Institutional Review Board of the Epidemiological and Clinical Research Information Network (JFMC-DB2020-01) and registered in the University Hospital Medical Information Network (UMIN000047044). The requirement for written informed consent was waived because of the retrospective nature of the study, and the opportunity to opt-out was provided. The authors did not access information that could identify individual participants.

### 2.2. Outcomes and Statistical Analysis

The relapse-free survival (RFS), OS, and disease-specific survival (DSS) were assessed. Patients’ baseline characteristics were summarized descriptively. Differences between treatment groups were determined using the chi-square test. To determine the prognosis after the three different treatments, we first matched the patients’ backgrounds using propensity score matching with the variables commonly used in these clinical trials: age, sex, tumor location (colon or rectum), extent of nodal dissection (D2 or D3), and depth of invasion (T1 or T2). Vascular and lymphatic invasion were not included as variables in the propensity score matching because these data were unavailable in some clinical trials. Likewise, histological differentiation grade (e.g., well, moderate, poor) was excluded due to its low prevalence. Direct-adjusted survival curves for OS, DFS, and DSS were delineated to reconcile the quality of surgery, staging, and treatment after recurrence between the past and present. The study period was stratified into two categories: JFMC7 plus JFMC15 and all remaining trials. This stratification was applied because only JFMC7 and JFMC15 included a surgery-alone arm, and the introduction of oxaliplatin was anticipated to exert a considerable influence on treatment outcomes. To further interpret the findings of this study, we conducted a multivariate analysis on the patient cohort prior to propensity score matching, with a primary focus on RFS as the key outcome in the present study, and incorporated histological differentiation, vascular invasion, and lymphatic invasion as variables. Hazard ratios (HRs) were calculated using the proportional hazards model with Firth’s method. Adjusted survival curves were generated using the “DIRADJ” option in the PROC PHREG statement of SAS software, ver. 9.4 (SAS Institute Inc., Cary, NC, USA), and edited using Photoshop CS2 (Adobe Systems, San Jose, CA, USA).

### 2.3. Generative Artificial Intelligence

During the revision of the manuscript, some sentences were translated into English using Google Translate, and the author thoroughly verified their accuracy and consistency. After revising the translated sentences, the English text was further proofread for grammar and flow using Microsoft Copilot, GPT-5 model (Microsoft Corporation, Redmond, WA, USA), and the author verified their accuracy and consistency again.

## 3. Results

### 3.1. Patient Characteristics

Of the 9867 patients, 609 (6.2%) had T1-2 stage III colorectal cancer. Five patients were excluded because of inadequate lymph node dissection. Thus, data from 604 patients with T1-2 stage III colorectal cancer was analyzed. After propensity score matching, 41 patients were included in each of the three groups (5FU, L-OHP, and surgery alone [surgery] groups) (Table 1 and Table 2). Before propensity score matching, there was a statistically significant difference in T stage (T1 or T2) (*p* < 0.01). However, after matching, no statistically significant differences remained in any variables. Treatment discontinuation occurred in 13 patients (31.7%) in the 5-FU group and in 7 patients (24.4%) in the L-OHP group. The median follow-up duration was >5 years.

### 3.2. Survival Analysis Before Propensity Score Matching

**Figure 1:**
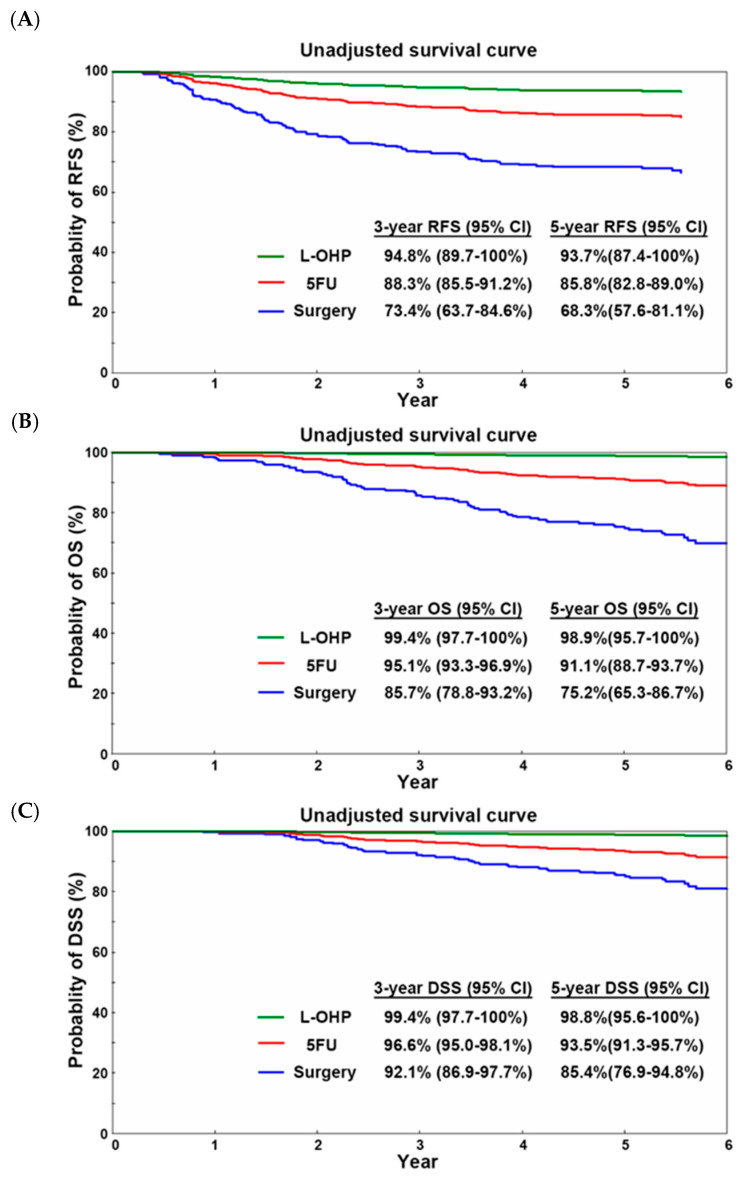
Survival curves of 604 patients before risk adjustment. (**A**) RFS in the surgery, L-OHP, and 5FU groups. (**B**) OS in the surgery, L-OHP, and 5FU groups. (**C**) DSS in the surgery alone, L-OHP, and 5FU groups.

Survival curves were delineated with the Breslow method using data from 604 patients. As shown in Figure 1 and Table 3, RFS significantly differed among the three groups, with better RFS in the 5FU and L-OHP groups than in the surgery group. The HR of RFS was 0.40 (95% confidence interval [CI]: 0.24–0.67, *p* < 0.01) and 0.17 (95% CI: 0.05–0.54, *p* < 0.01) in the 5FU and L-OHP groups, respectively. OS and DSS showed similar differences.

### 3.3. Analysis of Treatment Efficacy After Propensity Score Matching with Consideration of the Study Period

**Figure 2:**
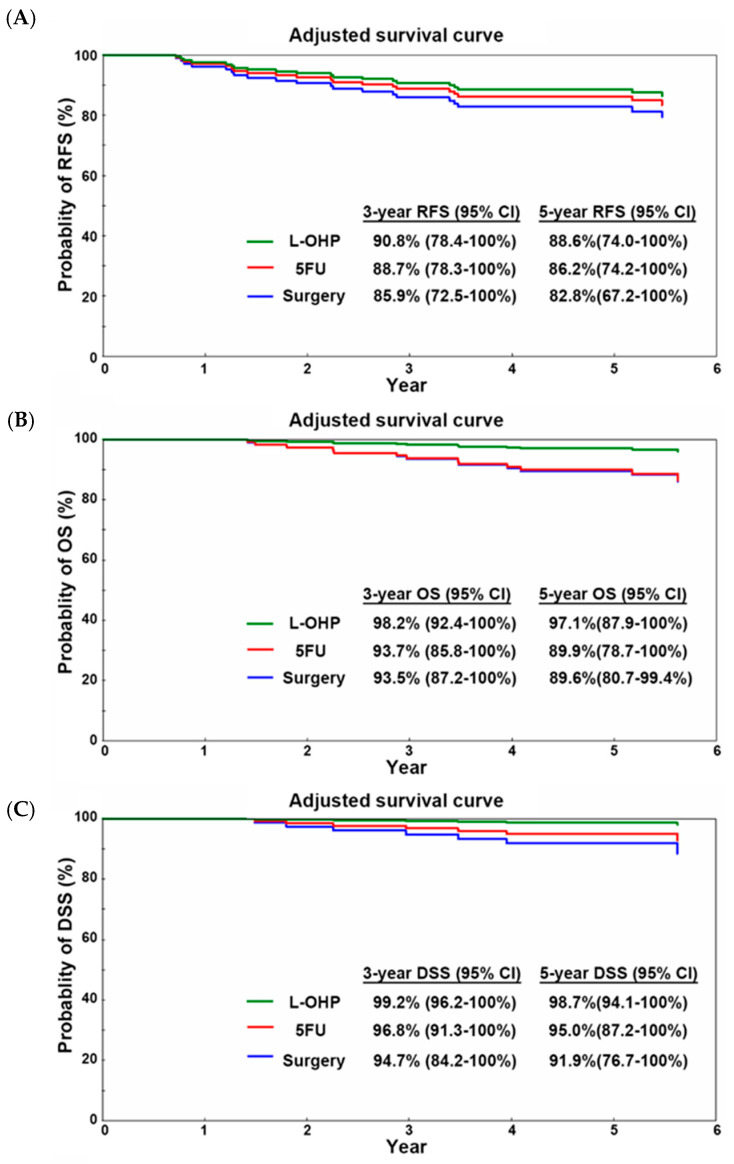
Survival curves of 143 patients after risk adjustment. (**A**) RFS in the surgery, L-OHP, and 5FU groups. (**B**) OS in the surgery, L-OHP, and 5FU groups. (**C**) DSS in the surgery, L-OHP, and 5FU groups.

After adjusting for patient factors, RFS was evaluated considering the study period (Table 3 and Figure 2). The HR of RFS was 0.79 (95% CI: 0.13–4.65, *p* = 0.79) and 0.64 (95% CI: 0.06–6.40, *p* = 0.70) in the 5FU and L-OHP groups, respectively. The 5-year RFS rate estimated using the Breslow method was 82.8% (95% CI: 67.2–100%), 86.2% (95% CI: 74.2–100%), and 88.6% (95% CI: 74.0–100%) in the surgery, 5FU, and L-OHP groups, respectively (Table 3 and Figure 2). The evaluation of OS followed a similar trend. The HR of OS was 0.97 (95% CI: 0.15–6.08, *p* = 0.97) and 0.26 (95% CI: 0.01–11.57, *p* = 0.49) in the 5FU and L-OHP groups, respectively, without statistical significance. The 5-year OS rate was 89.6% (95% CI: 80.7–99.4%), 89.9% (95% CI: 78.7–100%), and 97.1% (95% CI: 87.9–100%) in the surgery, 5FU, and L-OHP groups, respectively.

DSS analysis also revealed the limited efficacy of adjuvant chemotherapy. The HR of DSS was 0.60 (95% CI: 0.02–16.15, *p* = 0.76) and 0.16 (95% CI: 0.00–18.99, *p* = 0.45) in the 5FU and L-OHP groups, respectively. The 5-year DSS rate estimated using the Breslow method was 91.9% (95% CI: 76.7–100%), 95.0% (95% CI: 87.2–100%), and 98.7% (95% CI: 94.1–100%) in the surgery, 5FU, and L-OHP groups, respectively.

### 3.4. Multivariate Analysis

To reinforce prior findings and enhance the interpretability of the results, we conducted a multivariate analysis on 604 patients comprising the cohort prior to propensity score matching (Table 4). Variables excluded from the propensity score matching process—namely histological differentiation (well and moderately differentiated: *n* = 574, 95.0%), vascular invasion (positive: *n* = 273, 45.2%), and lymphatic invasion (positive: *n* = 154, 25.2%)—were incorporated into the model, with RFS designated as the dependent variable. The analysis yielded a HR of 0.65 (95% CI: 0.35–1.23; *p* = 0.19) for 5-FU (vs. surgery alone) and 0.37 (95% CI: 0.10–1.34; *p* = 0.13) for L-OHP (vs. surgery alone), indicating a potential benefit of adjuvant chemotherapy, albeit without statistical significance. Furthermore, female sex (vs. male sex) (HR = 0.61; 95% CI: 0.40–0.93; *p* = 0.02) and rectal tumor location (vs. colon) (HR = 2.19; 95% CI: 1.40–3.43; *p* = 0.0006) emerged as independent predictors of RFS. Conversely, no significant association was observed between the extent of lymph node dissection and histological differentiation.

## 4. Discussion

The present study investigated whether administering adjuvant chemotherapy benefits patients with stage III colorectal cancer with T1 or T1-2 wall invasion. Before adjusting for clinical characteristics and the study period, we observed significant differences in RFS, OS, and DSS among the three treatment groups. However, these differences were not observed after adjusting for certain variables. The estimated 5-year RFS rate was 82.8%, 86.2%, and 88.6% in the surgery, 5FU, and L-OHP groups, respectively. And, the estimate 5-year OS rate was 89.6%, 89.9%, and 97.1% in the surgery, 5FU, and L-OHP groups, respectively. These results contradict those of a previous study that showed the profound prognostic benefit of adjuvant chemotherapy in patients with T1 stage III colon cancer [14].

Specifically, the researchers analyzed 3137 patients with T1N+ disease using data from the National Cancer Database (NCDB), of whom 70.6% (*n* = 2216) received chemotherapy [14]. To minimize confounding, propensity score matching was applied, yielding two cohorts of 921 patients each. The 5-year overall survival was significantly higher in the chemotherapy group (83.4%) than in the non-chemotherapy group (63.0%). Further analysis indicated that insurance status, race, facility type (academic vs. community), and Charlson comorbidity index were not independently associated with chemotherapy omission. A key limitation discussed by the authors was the absence of data on postoperative complications, such as surgical site infections. In contrast, the population in the present study was drawn from clinical trial participants, which may have contributed to greater homogeneity in baseline characteristics and a more modest improvement in outcomes.

In contrast, a recent retrospective analysis of 307 consecutive patients with T1-2 colorectal cancer reported no benefit from adjuvant chemotherapy [15]. After excluding possible stage migration with <12 lymph nodes, 179 patients with T1-2 N0 and 32 with T1-2 N1 colon cancer were identified in the study. They found no significant difference in prognosis between patients with T1-2 N0 and N1 colon cancer or in OS and DFS between patients with T1-2 N1 colon cancer who received FOLFOX chemotherapy or not. They reported 5-year OS rates of >90% in all three groups and indicated that a different prognosis could not be expected with adjuvant chemotherapy in patients with T1-2 colon cancer.

Recently, a study has proposed a predictive model to estimate the benefit of adjuvant chemotherapy for stage III subgroups stratified by T and N factors [26], using data from the IDEA (International Duration Evaluation of Adjuvant Chemotherapy) study [27]. The IDEA study evaluated the clinical efficacy of 6-month versus 3-month oxaliplatin-based adjuvant chemotherapy. According to the method of the study, by integrating the findings of IDEA study with previous studies comparing surgery alone and 5-FU monotherapy and studies comparing doublet versus 5FU, a prognostic model for the benefit of 5-FU monotherapy and doublet adjuvant chemotherapy was developed. This calculation is based on the assumption that “HR is a reliable descriptor of the effect of each treatment across prognostic strata [26]”. The results indicated that T1 stage III colon cancer demonstrated substantially less benefit compared with T3/T4 stage III disease. Specifically, in T1N1a patients, surgery alone yielded a 5-year DFS of 79.6%, while 5-FU improved DFS by 5.6%, and the addition of 6 months of oxaliplatin provided a further 3.1% improvement. Conversely, in T4N1a patients, 5-FU improved DFS by approximately 12.1%, and adding 6 months of oxaliplatin resulted in an additional 7.4% improvement. The results of the present study were similar to those predictions, suggesting that the two studies reinforce each other.

Adjuvant chemotherapy improves recurrence rate and OS after curative resection of stage III colon and rectal cancers. L-OHP further improves OS, with effects persisting for >5 years [4,28]. L-OHP is a standard adjuvant treatment for resected stage III colon cancer; however, long-lasting peripheral neuropathy and other adverse reactions are significant problems [29,30], especially when the expected benefit is small. Therefore, the ‘Clinical Question 15’ of The Japanese Society for Cancer of the Colon and Rectum guidelines issued in 2019 stated that the selection of the treatment regimen should be balanced between anticipated baseline recurrence/morbidity and expected effects, considering related factors such as general condition and patient preference [31]. The guideline suggests that fluoropyrimidine-based chemotherapy is an active treatment option, particularly for patients with a low risk of recurrence. The recommendation for adjuvant chemotherapy with 5FU remains in the 2024 guidelines, indicating that there is still scope for its use [32]. The statement regarding the choice of regimen for low-risk patients is not supported by sufficient evidence; however, the results of the present and recent retrospective studies support this recommendation.

In the multivariate analysis of the present study, adjuvant chemotherapy appeared to be associated with a favorable prognosis; however, this association did not reach statistical significance. The HRs for RFS were 0.65 (95% CI: 0.35–1.23; *p* = 0.19) for 5-FU and 0.37 (95% CI: 0.10–1.34; *p* = 0.13) for L-OHP, compared with surgery alone. These findings align with previous analyses using propensity score matching and adjustment for study period, reinforcing the results. Gender and tumor location were identified as risk factors for recurrence, although their contributions have not been fully elucidated [33,34]. Because identifying high-risk groups may help guide adjuvant therapy selection, further research on these factors is warranted. Conversely, our findings indicate that the extent of lymph node dissection may not have a significant impact on clinical outcomes in patients with T1 or T2 stage III colorectal cancer, which contrasts with the results observed in patients with T3 or T4 colon cancer [25].

Compared with previous studies that used a nationwide database or single-center experience, the present study used individual data obtained from clinical trials, which is a strength of this study. However, this study has some limitations. First is the lack of information regarding the details of the pathological examination and the number of retrieved lymph nodes [35]. The second limitation is the inclusion of patients with rectal cancer. The treatment of rectal cancer is rapidly evolving, and the effects of neoadjuvant chemotherapy (preoperative chemoradiation was not a common practice in Japan during the study period) or lateral lymph node dissection [36,37] could not be assessed in this study. However, this study focused on T1-2 rectal cancer, and in patients with preoperative T1-2N0 disease, surgical resection remains one of the primary treatment options [10]. If lymph node metastasis is subsequently detected, chemotherapy is then considered. Although the proportion of such patients is thought to be small, this study may still provide valuable insights to support clinical decision-making in this scenario. Third, the sample size was small. T1-2 node-positive colorectal cancer accounts for a small proportion of cases of curatively resected colorectal cancers [13], partly explaining the paucity of trials focusing on patients with T1-2 stage III colorectal cancer. Additionally, a large number of patients is required to prove a statistical difference for a slight change in absolute survival [38]. For example, other researchers analyzed 1780 patients from the Surveillance, Epidemiology, and End Results database to determine whether adjuvant chemotherapy was effective for T1 node-positive colon cancer and found an incremental increase in DSS by 3.6%. Despite the anticipated difficulties in conducting this study, we agree that a reasonably large-scale, well-designed clinical study is required [16] to facilitate decision-making.

The fourth limitation is that this study relies on previous clinical trials; therefore, it lacks information on genomic features of tumors such as microsatellite instability (MSI), RAS mutation, BRAF mutation, and PD-L1 expression. For instance, regimens containing oxaliplatin have been shown to be effective in MSI-high colon cancers, whereas 5-FU alone has little effect in reducing the risk of recurrence [39]. In addition, biomarkers such as RAS mutation, BRAF mutation, and PD-L1 expression are actively being investigated in relation to treatment options and prognosis in many gastrointestinal cancers, including colorectal cancer [40,41,42]. Incorporating these genomic features into the present analysis might have led to more accurate results. Furthermore, evaluation using circulating tumor DNA (ctDNA) has also attracted increasing attention. A study of 1039 patients with stage II–IV CRC after radical resection demonstrated that ctDNA positivity at 4 weeks post-surgery was associated with a significantly higher risk of recurrence [43]. Positive ctDNA status has also been shown to predict greater benefit from adjuvant chemotherapy. Therefore, ctDNA analysis may help identify patients who should be recommended for chemotherapy, particularly those with T1-2 stage III colorectal cancer.

## 5. Conclusions

This study showed no significant improvement in prognosis after adjuvant chemotherapy in patients with T1-2 stage III colorectal cancer. The potential increase in 5-year RFS by 3.4% (5FU) and 5.8% (L-OHP) is consistent with the findings of recent studies, and the provision of adjuvant chemotherapy should be balanced with adverse reactions and patients’ preferences.

## Figures and Tables

**Table 1:** Patient characteristics before matching.

		Number and Proportion				Standardized Difference			*p* Value
		Surgery Alone (*n* = 57)	5FU (*n* = 486)	L-OHP (*n* = 61)	Total (*n* = 604)	Surgery Alone vs. 5FU	Surgery Alone vs. L-OHP	5FU vs. L-OHP	
Sex									
	male	29 (50.9%)	255 (52.5%)	26 (42.6%)	310 (51.3%)	0.03	0.17	0.20	0.35
	female	28 (49.1%)	231 (47.5%)	35 (57.4%)	294 (48.7%)				
Age (year)									
	<70	50 (87.7%)	370 (76.1%)	50 (82.0%)	470 (77.8%)	0.30	0.16	0.14	0.10
	≥70	7 (12.3%)	116 (23.9%)	11 (18.0%)	134 (22.2%)				
Tumor location									
	colon	25 (43.9%)	271 (55.8%)	40 (65.6%)	336 (55.6%)	0.24	0.45	0.20	0.06
	rectum	32 (56.1%)	215 (44.2%)	21 (34.4%)	268 (44.4%)				
T stage									
	T1	5 (8.8%)	158 (32.5%)	27 (44.3%)	190 (31.5%)	0.61	0.88	0.24	<0.01
	T2	52 (91.2%)	328 (67.5%)	34 (55.7%)	414 (68.5%)				
Lymph node dissection									
	D2	27 (47.4%)	220 (45.3%)	27 (44.3%)	274 (45.4%)	0.04	0.06	0.02	0.94
	D3	30 (52.6%)	266 (54.7%)	34 (55.7%)	330 (54.6%)				

**Table 2:** Patient characteristics after matching.

		Number and Proportion				Standardized Difference			*p* Value
		Surgery Alone (*n* = 41)	5-FU (*n* = 41)	L-OHP (*n* = 41)	Total (*n* = 123)	Surgery Alone vs. 5FU	Surgery Alone vs. L-OHP	5FU vs. L-OHP	
Sex									
	male	17 (41.5%)	17 (41.5%)	15 (36.6%)	49 (39.8%)	0.00	0.10	0.10	0.87
	female	24 (58.5%)	24 (58.5%)	26 (63.4%)	74 (60.2%)				
Age (year)									
	<70	34 (82.9%)	35 (85.4%)	36 (87.8%)	105 (85.4%)	0.07	0.14	0.07	0.82
	≥70	7 (17.1%)	6 (14.6%)	5 (12.2%)	18 (14.6%)				
Tumor location									
	colon	23 (56.1%)	22 (53.7%)	23 (56.1%)	68 (55.3%)	0.05	0.00	0.05	0.97
	rectum	18 (43.9%)	19 (46.3%)	18 (43.9%)	55 (44.7%)				
T stage									
	T1	5 (12.2%)	6 (14.6%)	7 (17.1%)	18 (14.6%)	0.07	0.14	0.07	0.82
	T2	36 (87.8%)	35 (85.4%)	34 (82.9%)	105 (85.4%)				
Lymph node dissection									
	D2	20 (48.8%)	19 (46.3%)	21 (51.2%)	60 (48.8%)	0.05	0.05	0.10	0.91
	D3	21 (51.2%)	22 (53.7%)	20 (48.8%)	63 (51.2%)				

**Table 3:** Hazard ratios of RFS, OS, and DSS.

		HR (95% Confidence Interval)	*p* Value
Pre-matching			
RFS (vs. surgery alone)			
	L-OHP	0.17 (0.05–0.54)	<0.01
	5FU	0.40 (0.24–0.67)	<0.01
OS (vs. surgery alone)			
	L-OHP	0.04 (0.00–0.71)	0.03
	5FU	0.33 (0.19–0.57)	<0.01
DSS (vs. surgery alone)			
	L-OHP	0.07 (0.00–1.37)	0.08
	5FU	0.43 (0.21–0.88)	0.02
Post-matching			
RFS (vs. surgery alone)			
	L-OHP	0.64 (0.06–6.40)	0.70
	5FU	0.79 (0.13–4.65)	0.79
OS (vs. surgery alone)			
	L-OHP	0.26 (0.01–11.57)	0.49
	5FU	0.97 (0.15–6.08)	0.97
DSS (vs. surgery alone)			
	L-OHP	0.16 (0.00–18.99)	0.45
	5FU	0.60 (0.02–16.15)	0.76

**Table 4:** Multivariate analysis of RFS with 604 patients.

Variables		HR (95% Confidence Interval)	*p* Value
Chemotherapy	5-FU vs. Surgery alone	0.65 (0.35–1.23)	0.19
	L-OHP vs. Surgery alone	0.37 (0.10–1.34)	0.13
Sex	Female vs. male	0.61 (0.40–0.93)	0.02
Age	70 or above or 70>	1.46 (0.89–2.39)	0.13
Location	Rectum vs. Colon	2.19 (1.40–3.43)	<0.01
Histology	Other vs. Well-Mod	0.73 (0.25–2.16)	0.57
T stage	T2 vs. T1	1.39 (0.81–2.41)	0.24
Lymph node dissection	D3 vs. D2	0.79 (0.52–1.21)	0.28
Study period	j33-41 vs. j07-15	0.61 (0.34–1.10)	0.10
Lymphatic invasion	ly(+)/ly(−) + unknown	1.04 (0.63–1.71)	0.88
Venous invasion	v(+)/v(−) + unknown	1.11 (0.65–1.92)	0.70

## Data Availability

Data is unavailable due to ethical restrictions.

## Abbreviations

The following abbreviations are used in this manuscript:

5FU	Fluorouracil
CI	confidence interval
DFS	disease-free survival
DSS	disease-specific survival
HRs	Hazard ratios
JFMC	Japanese Foundation for Multidisciplinary Treatment of Cancer
L-OHP	oxaliplatin
OS	overall survival
RFS	relapse-free survival
